# Diagnostic Yield of Dental Radiography and Cone-Beam Computed Tomography for the Identification of Anatomic Structures in Cats

**DOI:** 10.3389/fvets.2019.00058

**Published:** 2019-02-28

**Authors:** Colleen M. Heney, Boaz Arzi, Philip H. Kass, David C. Hatcher, Frank J. M. Verstraete

**Affiliations:** ^1^Dentistry and Oral Surgery Service, William Pritchard Veterinary Medical Teaching Hospital, School of Veterinary Medicine, University of California, Davis, Davis, CA, United States; ^2^Department of Surgical and Radiological Sciences, School of Veterinary Medicine, University of California, Davis, Davis, CA, United States; ^3^Department of Population Health and Reproduction School of Veterinary Medicine, University of California, Davis, Davis, CA, United States; ^4^Diagnostic Dental Imaging Center, Sacramento, CA, United States

**Keywords:** oral anatomy, cats, CBCT, cone-beam computed tomography, dentition, dental radiography

## Abstract

The objective of this study was to evaluate the diagnostic yield of dental radiography (DR) and 3 cone-beam computed tomography (CBCT) methods for the identification of predefined anatomic structures in cats. For 5 feline cadaver heads and 22 client-owned cats admitted for evaluation and treatment of dental disease, a total of 22 predefined anatomic structures were evaluated separately by use of the DR method and 3 CBCT software modules [multiplanar reconstructions (MPR), tridimensional (3-D) rendering, and reconstructed panoramic views (Pano)]. A semi quantitative scoring system was used, and mean scores were calculated for each anatomic structure and imaging method. The Friedman test was used to evaluate values for significant differences in diagnostic yield. For values that were significant the Wilcoxon signed rank test was used with the Bonferroni-Holm multiple comparison adjustment to determine significant differences among each of the possible pairs of diagnostic methods. Differences of diagnostic yield among the DR and 3 CBCT methods were significant for 17 of 22 anatomic structures. For these structures, DR scores were significantly higher than scores for Pano views for 2 of 17 structures, but DR scores were significantly lower than scores for Pano views for 6 anatomic structures, tridimensional rendering for 10 anatomic structures, and MPR for 17 anatomic structures. In conclusion, it was found that CBCT methods were better suited than DR for the identification of anatomic structures in cats. Results of this study can serve as a basis for CBCT evaluation of dentoalveolar and other maxillofacial bony lesions in cats.

## Introduction

The benefits of performing dental radiography (DR) are well-documented, and its routine use has been considered standard of practice in veterinary dentistry for the past 20 years (1, 2). Full-mouth DR (i.e., 10 standard projections combining intraoral and extraoral techniques) represent the current diagnostic criterion-referenced standard in feline veterinary dentistry (3). Dental radiography is recognized as a valuable imaging modality with high diagnostic yield, which can be implemented at a relatively low cost. However, the nature of creating a two-dimensional (2-D) image of a tridimensional (3-D) structure can lead to inherent difficulties in image interpretation. Projection errors due to subtle variations in angulation of the x-ray beam, and errors in identification of anatomic structures due to superimposition may occur with the use of DR (4).

Cone-beam computed tomography (CBCT) is rapidly gaining acceptance in the field of veterinary dentistry and oral surgery. In human dentistry, CBCT is used when conventional DR cannot supply satisfactory diagnostic information. Cone-beam CT may prove to be the next major advancement in veterinary dentoalveolar and maxillofacial imaging because of its ability to provide 3-D imaging at a lower cost than multidetector row CT (i.e., conventional CT), and at a lower radiation risk comparable to conventional CT. The use of rapid scan technology, which allows for faster image acquisition than conventional CT, and the ability to post process the volumetric data into various 2-D and 3-D reconstructions, makes CBCT an attractive imaging modality. Currently, validation of the clinical application of CBCT in veterinary patients is ongoing (5–12), and more research is required to validate the use of CBCT to responsibly promote its use in routine veterinary clinical practice.

The objective of the present study was to evaluate the ability of conventional full-mouth DR and CBCT to identify predefined clinically relevant anatomic structures of the orofacial region in cats. For this purpose, 22 predefined anatomic structures were evaluated by use of both imaging modalities. Furthermore, to characterize the diagnostic method that was most useful among the CBCT methods available, 3 software modules provided by specialized software were evaluated separately. We hypothesized that CBCT images would yield more detailed information and would be better suited than DR for use in identifying anatomic structures in cats.

## Materials and Methods

### Animals

Five young adult, non-brachycephalic, cadaver heads with complete adult dentition, of unknown breed and sex were evaluated first to calibrate the primary observer (CH) in image acquisition and interpretation in addition to establishing a baseline for normal anatomy. These cats were euthanized for reasons unrelated to this study. Client-owned cats that were admitted to the Dentistry and Oral Surgery Service at the University of California-Davis for evaluation and treatment of oral disease between August 2014 and February 2017 for which full-mouth DR and CBCT scans of the skull were obtained were included in the study. Informed consent was obtained from each owner, and the study was approved by the AAALAC accredited University of California-Davis Institutional Animal Care and Use Committee and the Clinical Trials Review Board.

### Image Acquisition

Dental radiography and CBCT were performed on the cadaver heads. Client-owned cats were anesthetized, and DR and CBCT were performed. Full-mouth dental radiographs were obtained by use of a digital intraoral imaging system (Heliodent MD, Siemens Sirona; ScanX, Air Techniques) at 60 kVp, 7 mA, and exposure time of 0.12–0.20 s (depending on location of the evaluated teeth). This system yielded a resolution of up to 18 linepairs/mm, which equated to a pixel size of 55.5 μm. Radiographic images included the standard series of views in accordance with American Veterinary Dental College guidelines (3). A CBCT unit (NewTom 5G CBCT scanner, NewTom) was used to obtain images. Field of view was 15 × 12 cm, and serial slices of the skull were obtained with a scan time of 24 s, which resulted in a voxel size (slice thickness) of 150 μm. All skulls were scanned in sternal recumbency with the mouth propped open slightly with 2 × 2 inch gauze.

### Image Evaluation and Scoring

**Figure 1:**
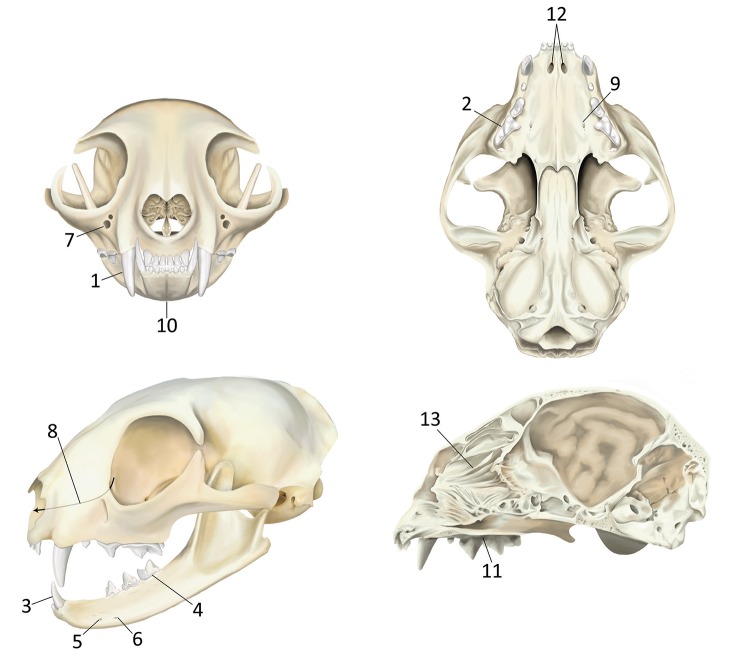
Predefined anatomic structures evaluated in cats by use of DR and CBCT images for each of 3 software modules. (1) Maxillary canine tooth dentoalveolar structures; (2) Maxillary fourth premolar tooth dentoalveolar structures; (3) Mandibular canine tooth dentoalveolar structures; (4) Mandibular first molar tooth dentoalveolar structures; (5) Middle mental foramena; (6) Caudal mental foramena; (7) Infraorbital foramena; (8) Nasolacrimal canals; (9) Major palatine foramena; (10) Mandibular symphysis; (11) Plane of the hard palate; (12) Palatine fissures; (13) Nasal turbinates. All anatomic locations were evaluated bilaterally where applicable.

Dental radiography (DR method) and 3 CBCT software modules [reconstructed panoramic views (Pano method), tridimensional rendering (3-D method), and serial CBCT slices and multiplanar reconstructions (MPR method)] were evaluated separately for their usefulness in identification of 22 predefined clinically relevant oral anatomic structures (Figure 1). Images were examined on medical grade flat-screen monitors (ASUS PB278Q, ASUSTeK Computer Inc.) by use of commercially available specialized software (for DR: Metron-Dental 7.40.34.0, Epona Tech LCC; for CBCT: Anatomage *Invivo5*; Anatomage Inc.), and each method was scored separately for each structure by 1 observer (CH) who was trained and calibrated in image acquisition and interpretation by 2 board-certified veterinary dentists (BA and FJMV) and a board-certified human oral radiologist (DCH).

**Figure 2:**
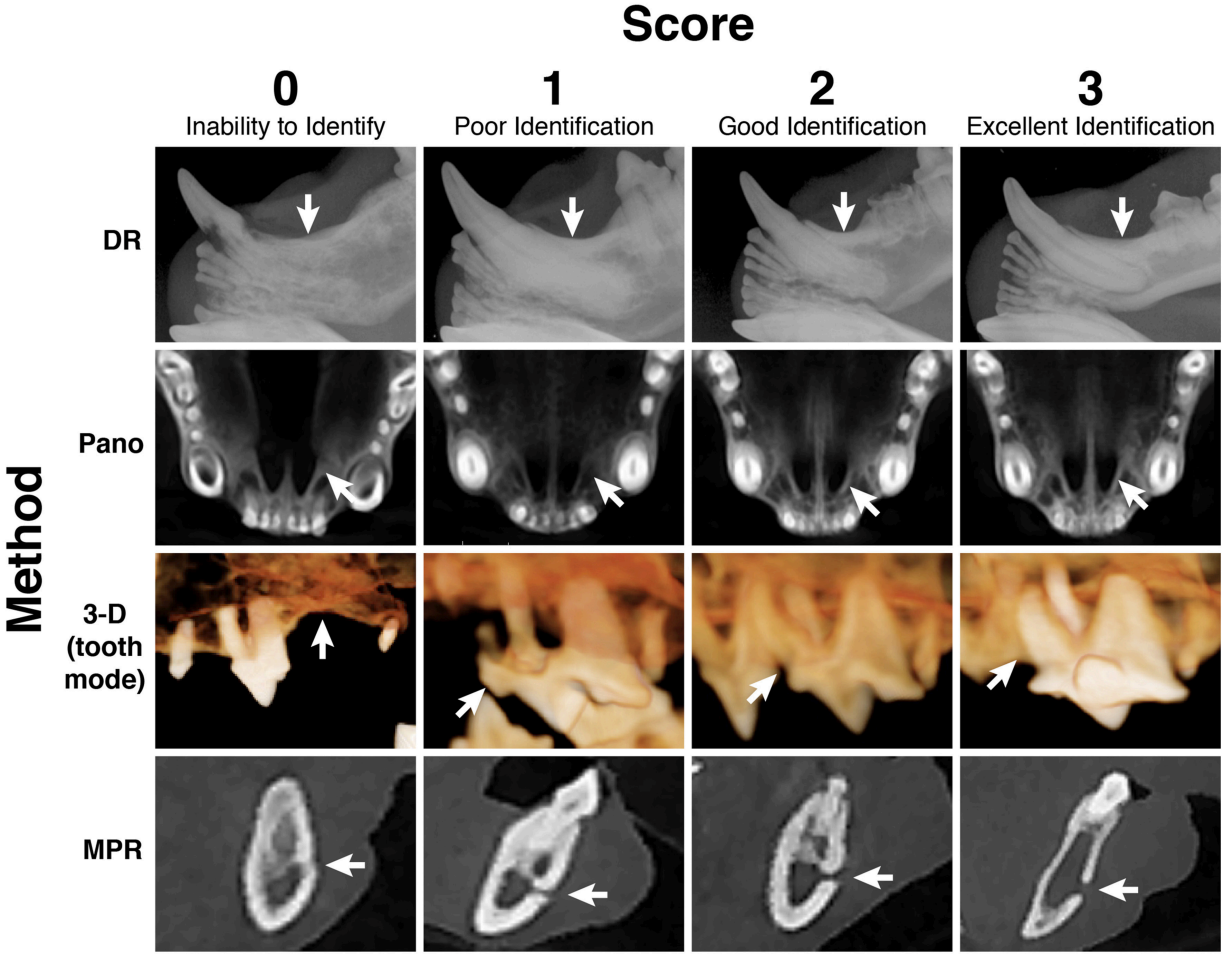
A semi-quantitative scoring system on a scale of 0–3 was designed to assign a numerical value to every anatomic structure for every head and for all 4 imaging methods. Displayed are examples of all 4 numerical scores, for 4 separate anatomic structures, evaluated in each of the 4 imaging methods, respectively. Score 0 = inability to identify the anatomic structure; Score 1 = poor identification of anatomic structure; Score 2 = good identification of anatomic structure; Score 3 = excellent identification of anatomic structure; DR method for evaluation of the left mandibular canine tooth dentoalveolar structure; Pano method for evaluation of the palatine fissures; 3-D method in tooth mode for evaluation of the left maxillary fourth premolar tooth dentoalveolar structure; MPR method for evaluation of the left caudal mental foramen.

A semi quantitative scoring system was used for each imaging method. Scoring was on a scale of 0–3 as follows: 0 = inability to identify the anatomic structure, 1 = poor identification of anatomic structure, 2 = good identification of anatomic structure, and 3 = excellent identification of anatomic structure (Figure 2). Mean score for each anatomic structure was calculated for each imaging method. Findings for all 4 methods were recorded separately without reference to each patient's medical record to limit biased interpretation for each finding. Mean score of a method was calculated for each landmark and as a total for each imaging method and were reported as poor (mean score <1), moderate (mean score ≥1 and <2), good (mean score ≥2 and <3), and excellent (mean score = 3).

### MPR Method

Each skull was oriented by use of the specialized software (Anatomage *Invivo5*; Anatomage Inc.) to properly align the Cartesian coordinate systems of the CBCT scanner to the sagittal, transverse, and dorsal planes of the patient. The anatomic landmarks used to standardize alignment were the temporomandibular joints, the plane of the hard palate, and the midline suture of the skull. Thus, each point of the skull was assigned a specific position within the 3-D space by use of 3 coordinates that could then be recognized by the CBCT software for further image manipulation. The sagittal, transverse, and dorsal slices in combination with custom cross-sections were evaluated with the preset software settings for dental use and hard sharpening of the contrast.

### 3-D Method

**Figure 3:**
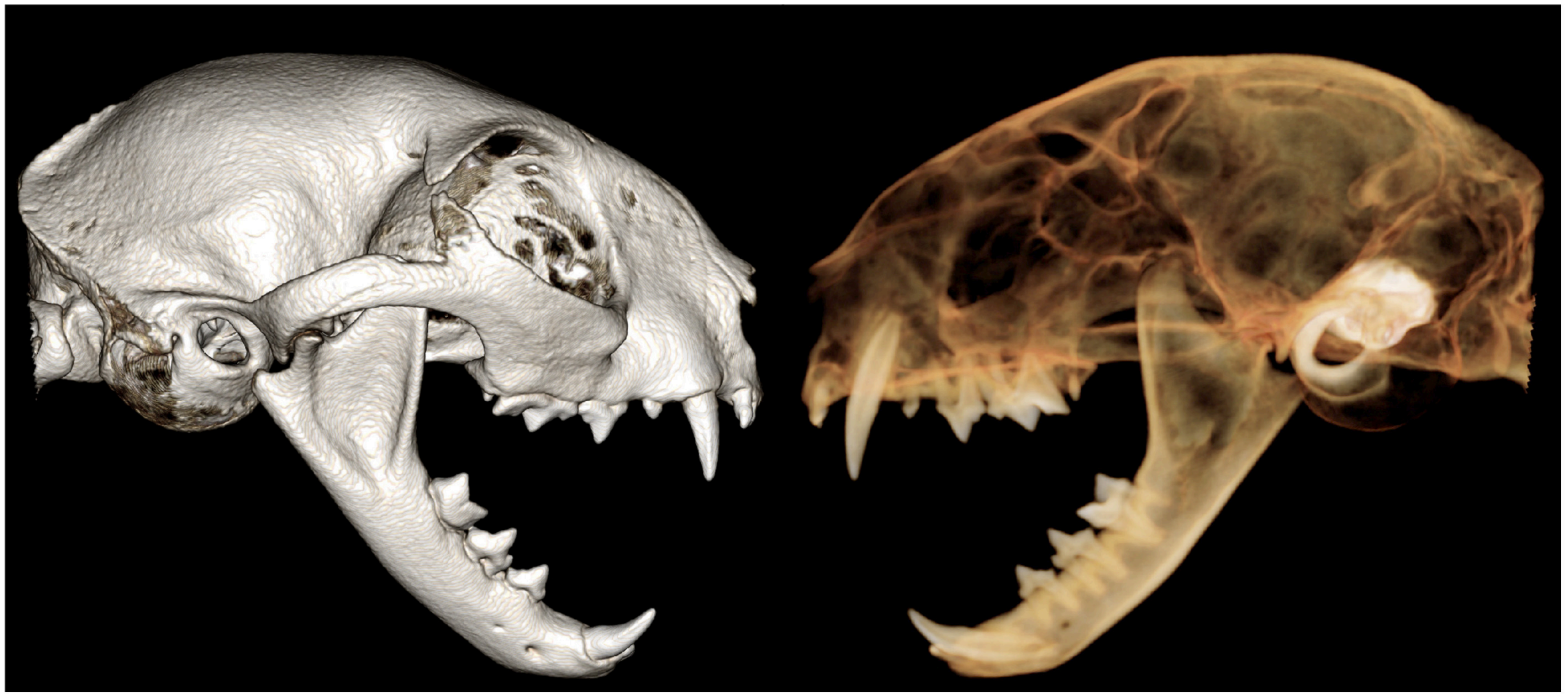
Tridimensional (3-D) rendering of the skull of the cat in bone **(Left)** and tooth **(Right)** mode. Notice that tooth roots are visible only in the tooth mode, whereas other anatomic structures are more visible in bone mode.

Tridimensional rendering is a volume-rendering technique whereby the entire volume of a CBCT scan is composed into 1 block of data, which can be selectively displayed. The volume-rendering algorithm used involved all acquired data and assigned voxels to various colors and transparency values (based on their attenuation values) to enhance discrimination among structures. The volume-rendering technique allows users to adjust the display characteristics of selected tissue types that have unique x-ray attenuation values. The software used for the study here provided 2 main settings (tooth and bone mode; Figure 3). To optimize and standardize evaluation of the skulls for the tooth mode, images were set to level/brightness of 2,000/0, opacity was set to 1/3, and the window/contrast was decreased to 4,600/−0.07. For the bone mode, settings were adjusted to a level/brightness window in which the density for oral soft tissues was just excluded from being shown [level (or brightness) = 1,600–1,400 (or 0.10–0.15)], opacity was set to 7/8, and window and contrast were increased to 2,400 and 0.20. The clipping tool was used to evaluate the right and left sides of the skull as well as to separately evaluate the lower jaw and upper jaw. Incrementally advancing deeper into the skull by removing (i.e., clipping away) each slice was not performed. The 3-D images were rotated to enable evaluation of each skull from all sites and angles in both bone and tooth modes.

### Pano Method

**Figure 4:**
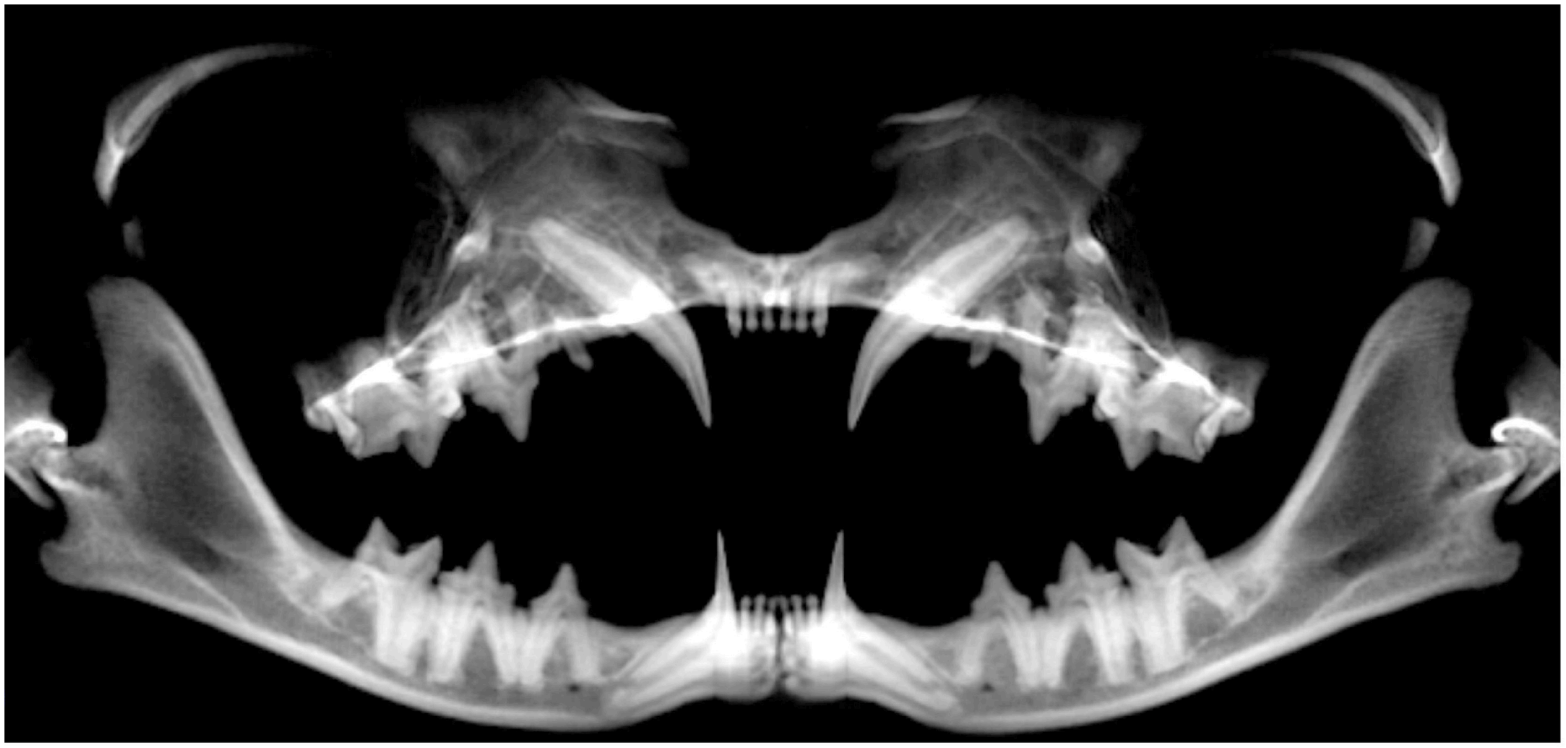
Standard panoramic view of the skull of a cat. Notice the inability to exploit the full potential of lateral radiographs for evaluation of anatomic structures, particularly in the regions of maxillary incisor teeth and the mandibular incisor and canine teeth.

**Figure 5:**
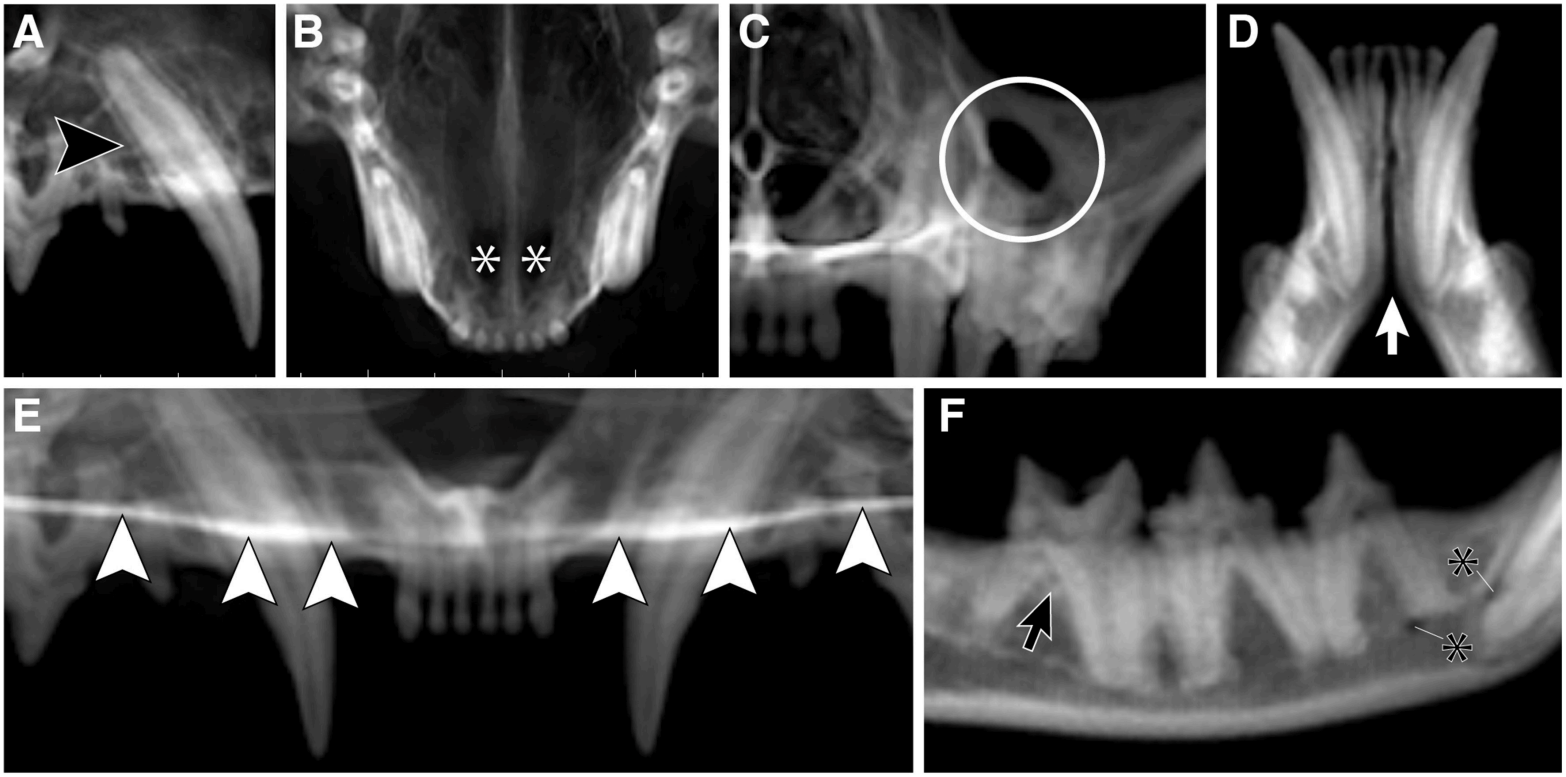
The CBCT optimized reconstructed panoramic (Pano) views for the right maxillary canine tooth (black arrowhead; **A**), palatine fissures (white asterisks; **B**), left infraorbital foramen (white circle; **C**), mandibular symphysis (white arrow; **D**), plane of the hard palate (white arrowheads; **E**), and right middle and caudal mental foramina (black asterisks; **F**), and left mandibular first molar tooth (black arrow; **F**) in a cat.

Because of the configuration of cat skulls, the standard panoramic view was not sufficient for evaluation of the orofacial anatomy (Figure 4). Instead, orientation of the skull was adjusted accordingly, and multiple Pano views with optimized plane location, shape, and thickness were created to enable us to obtain the full benefit of truly parallel radiographs (Figure 5).

### Statistical Analysis

Descriptive statistics and mean scores were reported as mean ± SD. Scores from all patients for each anatomical structure and each imaging method were used to calculate the overall mean ± SD. The Friedman test was used to evaluate differences in diagnostic yield of the ranked scores for the DR and the 3 CBCT methods. When this test lead to the finding of significant differences between at least 2 methods, the Wilcoxon signed rank test was used with the Bonferroni–Holm multiple comparisons adjustment to determine significant differences among each of the 6 possible pairs of diagnostic methods. Significance was set at values of *P* < 0.05.

## Results

### Animals

In addition to the 5 cadaver heads initially evaluated, 22 client-owned cats [17 males (16 castrated and 1 sexually intact] and 5 females (5 spayed and 0 sexually intact)] were included in the study. Breeds included domestic shorthair (*n* = 9), domestic medium hair (2), domestic longhair (3), Burmese (3), Bengal (1), British shorthair (2), Scottish fold (1), and Siamese mix (1). Mean ± SD age of the cats was 5.9 ± 3.7 years (range, 5 months−12 years), and mean body weight was 5.0 ± 1.1 kg (range, 3.3–7.2 kg). In addition, data collected from the 5 cadaver heads were included in the statistical analysis.

### Overall Scores

**Figure 6:**
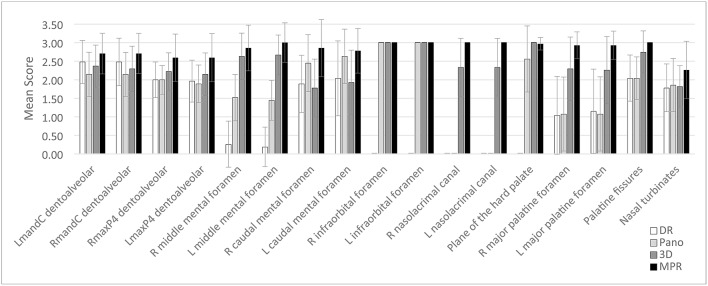
Mean score and standard deviation for each of 17 anatomic structures with at least 2 methods that differed significantly (*P* < 0.05) among the DR method (white bars) and the 3 CBCT methods [Pano method (light gray bars), 3-D method (dark gray bars), and MPR method (black bars)] for evaluation of cats. Scores were assigned by use of a scale of 0–3 as follows: 0 = inability to identify the anatomic structure, 1 = poor identification of anatomic structure, 2 = good identification of anatomic structure, and 3 = excellent identification of anatomic structure.

Mean scores for each anatomic structure and each imaging method are reported in Figure 6. Of the 22 predefined oral anatomic structures, 5 structures were excluded from further statistical analysis because of a lack of significant differences among the imaging methods, as confirmed by results of the Friedman test. The 5 excluded structures were the right and left maxillary canine teeth, the right and left mandibular first molar teeth, and the mandibular symphysis. Statistical analysis for all combination pairs among the DR method and 3 software modules for the 17 remaining anatomic structures were compared and depicted in Table 1.

**Table 1:** Results of the Wilcoxon signed rank test for all 6 possible pairs of diagnostic methods for evaluation of anatomic structures in the skulls of cats.

**Variable**	**DR vs. Pano**	**DR vs. 3-D**	**DR vs. MPR**	**Pano vs. 3-D**	**Pano vs. MPR**	**3-D vs. MPR**
LmandC dentoalveolar structure	0.003^*^	0.257	0.014^*^	0.126	<0.001^*^	0.003^*^
RmandC dentoalveolar structure	0.003^*^	0.025^*^	0.014^*^	0.263	<0.001^*^	0.003^*^
RmaxP4 dentoalveolar structure	1.000	0.026	0.002^*^	0.014^*^	0.001^*^	0.011^*^
LmaxP4 dentoalveolar structure	0.414	0.099	<0.001^*^	0.008^*^	<0.001^*^	0.002^*^
R middle mental foramen	<0.001^*^	<0.001^*^	<0.001^*^	0.003^*^	<0.001^*^	0.169
L middle mental foramen	<0.001^*^	<0.001^*^	<0.001^*^	<0.001^*^	<0.001^*^	0.015^*^
R caudal mental foramen	0.038	0.059	<0.001^*^	0.015	0.047	<0.001^*^
L caudal mental foramen	0.011^*^	0.728	0.009^*^	0.004^*^	0.118	<0.001^*^
R infraorbital foramen	<0.001^*^	<0.001^*^	<0.001^*^	1.000	1.000	1.000
L infraorbital foramen	<0.001^*^	<0.001^*^	<0.001^*^	1.000	1.000	1.000
R nasolacrimal canal	1.000	<0.001^*^	<0.001^*^	<0.001^*^	<0.001^*^	0.001^*^
L nasolacrimal canal	1.000	<0.001^*^	<0.001^*^	<0.001^*^	<0.001^*^	0.001^*^
Plane of the hard palate	<0.001^*^	<0.001^*^	<0.001^*^	0.026	0.090	0.317
R major palatine foramen	0.976	<0.001^*^	<0.001^*^	<0.001^*^	<0.001^*^	0.012^*^
L major palatine foramen	0.757	<0.001^*^	0.001^*^	<0.001^*^	<0.001^*^	0.008^*^
Palatine fissures	1.000	0.001^*^	<0.001^*^	<0.001^*^	<0.001^*^	0.026
Nasal turbinates	0.696	0.564	0.009	0.787	0.021	0.010

The diagnostic methods were dental radiography (DR) and CBCT with 3 software modules [reconstructed panoramic views (Pano), tridimensional rendering (3-D), and custom multiplanar reconstructions (MPR)]. Results reported are P-values; values with

* *were considered significant at after multiple comparison adjustment*.

### DR Method

Compared with results for the Pano method, scores for the DR method were significantly higher for 2 of 17 anatomic structures, which represented 2 of the 8 dentoalveolar structures selected for identification (left and right mandibular canine tooth dentoalveolar structures). In addition, DR scores were higher, although not significantly, for the left maxillary fourth premolar dentoalveolar structure, and the left major palatine foramen. The DR scores were significantly lower for 10 anatomic structures, when compared with scores for the 3-D method and for all 17 anatomic structures when compared with scores for the MPR method. Mean DR score was poor for 7 anatomic structures, moderate for 5 anatomic structures, and good for 10 anatomic structures. Overall mean score for the DR method was 1.37, which indicated a moderate ability for use in identification of anatomic structures.

### MPR Method

For all 17 predefined anatomic structures with significant differences among methods, MPR scores were highest for all 17 of the anatomic structures, compared with scores for the DR, Pano, and 3-D methods. Compared with DR scores, MPR scores were significantly higher for all 17 anatomic structures. The MPR scores were significantly higher than Pano scores for the left and right mandibular canine teeth dentoalveolar structure, the left and right maxillary fourth premolar teeth dentoalveolar structure, the left and right middle mental foramen, the left and right nasolacrimal canal, the left and right major palatine foramen, and the palatine fissures, which represent 11 of the 17 anatomic structures. In addition, MPR scores were higher, although not significantly, for the left and right caudal mental foramen, the plane of the hard palate, and the nasal turbinates, compared with the Pano scores. The MPR scores were significantly higher than 3-D scores for the left and right mandibular canine dentoalveolar structure, the left and right maxillary fourth premolar dentoalveolar structure, the left middle mental foramen, the left and right caudal mental foramen, the left and right nasolacrimal canal, the left and right major palatine foramen, which represent 11 of the 17 anatomic structures. In addition, MPR scores were higher, although not significantly, for the right middle mental foramen, the palatine fissures, and the nasal turbinates, compared with the 3-D scores. The MPR scores were lower, although not significantly, for the plane of the hard palate, compared with the 3-D scores. Mean MPR score was moderate for 1 anatomic structure, good for 15 anatomic structures, and excellent for 6 anatomic structures. Overall mean score for the MPR method was 2.73, which indicated a good ability for use in identification of anatomic structures.

### 3-D Method

Scores for the 3-D method were significantly higher than scores for the DR method for the left and right middle mental foramen, the left and right infraorbital foramen, the left and right nasolacrimal canal, the plane of the hard palate, the left and right major palatine foramen, and the palatine fissures, which represented 10 of 17 anatomic structures. In addition, they were also higher, but not significantly so, for the left and right maxillary fourth premolar dentoalveolar structure, and the nasal turbinates, representing another 3 anatomic structures. The 3-D scores were lower for the left and right mandibular canine dentoalveolar structure, and the left and right caudal mental foramen.

In comparison to scores for the Pano method, 3-D scores were significantly higher in 9 of 17 anatomic structures; the left and right maxillary fourth premolar dentoalveolar structure, the left and right middle mental foramen, the left and right nasolacrimal canal, the left and right major palatine foramen, and the palatine fissures. In addition, they were also higher, but not significantly so, for the left and right mandibular canine dentoalveolar structure, and the plane of the hard palate, representing another 3 anatomic structures. The 3-D scores were significantly lower for the left caudal mental foramen, compared with Pano scores.

Scores for the 3-D method were significantly higher than scores for the MPR method in 11 of 17 anatomic structures; the left and right mandibular canine teeth dentoalveolar structure, the left and right maxillary fourth premolar teeth dentoalveolar structure, the left middle mental foramen, the left and right caudal mental foramen, the left and right nasolacrimal canal, and the left and right major palatine foramen. In addition, the scores were higher, although not significantly so, for the right middle mental foramen, the palatine fissures, and the nasal turbinates.

Mean 3-D score was moderate for 3 anatomic structures, good for 16 anatomic structures, and excellent for 3 anatomic structures. Overall mean score for the 3-D method was 2.34, which indicated a good ability for use in identification of anatomic structures.

### Pano Method

Scores for the Pano method were significantly higher than those for the DR method for the left and right middle mental foramen, the left caudal mental foramen, and the left and right infraorbital foramen, which represented 6 of 17 predefined anatomic structures. Scores for the Pano method were significantly higher than those for the 3-D method for the left caudal mental foramen. The Pano scores were significantly lower than scores for the 3-D method for 9 of 17 anatomic structures. In addition, Pano scores were higher, although not significantly, for the right caudal mental foramen, and the nasal turbinates, compared with the DR and 3-D scores. The Pano scores were lower for the left and right maxillary fourth premolar dentoalveolar structure, the left and right middle mental foramen, the left and right nasolacrimal canal, the left and right major palatine foramen, and the palatine fissures, compared with 3-D scores.

The Pano scores were significantly lower than scores for the MPR method for 11 of 17 anatomic structures. The Pano scores were lower for the left and right mandibular canine teeth dentoalveolar structure, the left and right maxillary fourth premolar teeth dentoalveolar structure, the left and right middle mental foramen, the left and right nasolacrimal canal, the left and right major palatine foramen, and the palatine fissures, compared with MPR scores. Mean Pano score was poor for 2 anatomic structures, moderate for 7 anatomic structures, good for 11 anatomic structures, and excellent for 2 anatomic structures. Overall mean score for the Pano method was 1.90, which indicated a moderate ability for use in identification of anatomic structures.

## Discussion

To our knowledge, this is the first study to investigate the diagnostic yield of CBCT compared to DR for the evaluation of oral and maxillofacial anatomic structures of cats. In the present study, we found several important and clinically relevant differences and similarities in diagnostic yield between the imaging methods. First, with the exception of the mandibular canine teeth dentoalveolar structure, the diagnostic yield of the Pano method was comparable to that of the DR method for dentoalveolar structures. Second, the combined diagnostic yield of 3 CBCT imaging modalities for non-dentoalveolar structures was superior than that of the DR method. Importantly, it was determined that there are differences in diagnostic yield between the 3 CBCT software modules. In addition, the 3-D method was shown to offer advantages for evaluation of skeletal structures over the DR method. Finally, the MPR method had the highest diagnostic yield as a single software module compared to all other software modules and DR.

The feasibility of dental panoramic imaging for dental arch evaluation in small animals has been previously investigated (5). In the present study, a single panoramic view was found to be unsuitable for evaluation of the entire skull of the cat due to the inability to include all anatomic structures of interest in a single curved plane without superimposition of anatomic structures. This finding was similar to that of a previous study that evaluated the panoramic method for use in evaluating the identification of anatomic structures in brachycephalic dogs (7). However, the use of multiple reconstructed panoramic views for evaluation of anatomic landmarks in cats allowed for true parallel imaging and for the elimination of overlapping structures. We also demonstrated that the diagnostic yield of the Pano method was comparable to that of the DR method for the evaluation of dentoalveolar structures. The diagnostic yield between the 2 methods for evaluation of the maxillary canine teeth, maxillary fourth premolar teeth, and mandibular first molar teeth dentoalveolar structures was comparable. The diagnostic yield was shown to be significantly higher for the DR method over the Pano method for the evaluation of the mandibular canine teeth dentoalveolar structure. However, the diagnostic yield of the MPR method for evaluation of the maxillary fourth premolar teeth and mandibular canine teeth was shown to be significantly higher than any of the other 3 imaging methods.

When evaluated together, the diagnostic yield of the 3 CBCT imaging modalities for the evaluation of non-dentoalveolar anatomic structures, such as canals and foramina, were superior to that of the DR method, except for the mandibular symphysis, which had comparable diagnostic yield between all imaging methods. This is consistent with previous studies in both veterinary (7, 13, 14) and human (4, 15, 16) dentistry that have shown that 3-D imaging is better suited to the identification and evaluation of anatomic structures than 2-D imaging such as dental radiography. The results of these studies, which showed that cephalometric evaluation of 2-D imaging often renders both inaccurate and imprecise measurements in comparison to 3-D imaging, encourage the exploration of potential further applications of CBCT in veterinary dentistry. The DR method and Pano method were unable to evaluate the nasolacrimal ducts, which is corroborated by a previous study that described the nasolacrimal system in brachycephalic cats (17). Additional studies in human dentistry have evaluated not only the ability to identify anatomic structures, but also the ability to determine accurate dimensions of those anatomic structures utilizing CBCT imaging (16, 18, 19). From a clinical perspective, having a precise imaging modality for determining the dimensions of anatomic structures such as the mandibular canal is valuable for fracture management and surgical planning for placement of internal fixation.

While it was determined that CBCT imaging modules, when evaluated together, were superior for most of the anatomic structures evaluated, it was also determined that there are significant differences in diagnostic yield between the 3 CBCT software modules for several anatomic structures. Not all CBCT viewing modalities were equal in their ability to identify various anatomic structures. This finding is considered clinically relevant, as it is important to take this into account in the development of standardized viewing protocols for CBCT studies of the skull in cats. Some of the CBCT viewing modalities, such as the 3-D method, anecdotally allow the evaluator to develop a general impression of a study in less time than systematically evaluating the study with other CBCT viewing modalities, like the MPR method. However, it is important to keep in mind that viewing a CBCT study utilizing any single modality may lead to missing clinically significant findings that can be identified via other viewing modalities. Proper evaluation of a CBCT study should be performed via a combined systematic review of all viewing modalities individually.

For the evaluation of skeletal structures, the diagnostic yield of the 3-D method was shown to be significantly higher than that of the DR method. The 3-D method and DR method were comparable in diagnostic yield for identification of all dentoalveolar structures evaluated, in addition to the caudal mental foramen and the nasal turbinates. However, the middle mental foramen, infraorbital foramen, nasolacrimal canals, plane of the hard palate, major palatine foramen, and palatine fissures were better evaluated by the 3-D method over the DR method. This was considered clinically important finding as it has implications for maxillofacial trauma management and surgical planning. This finding supports the notion that DR is inappropriate as a sole imaging modality for determining the structural integrity of the skeletal anatomic structures that may be damaged in clinical cases of maxillofacial trauma. The use of CBCT imaging via the 3-D viewing modality should be utilized in these cases for surgical planning and to assess outcomes.

Overall, as a single imaging method, the MPR method had the highest diagnostic yield compared to all other software modules and DR. When using tomographic slices, rather than images in which an entire volume is compressed into a 2-D image, the problem of superimposition of unrelated structures onto the anatomic structure of interest decreases. This is particularly problematic in the maxillary premolar and molar region of the cat, where the zygomatic arch can be superimposed over the roots of the dentoalveolar structures if imperfect radiographic technique is performed. Additionally, when using the MPR method appropriately, it is possible to evaluate each root separately. For these reasons, the superiority of MPR techniques over DR becomes obvious.

It was determined that CBCT obtains significantly more information than DR for identifying anatomic structures in cats. However, this study also shows that despite the overall superiority of CBCT methods, there is still considerable value in the use of dental radiography, as it can have comparable diagnostic yield for the identification of certain anatomic landmarks compared to that of CBCT (for the maxillary canine teeth and mandibular first molar teeth dentoalveolar structures, and for the mandibular symphysis). We attribute this largely to the superior image resolution of the DR method. However, for 17 of the 22 anatomic structures evaluated in this study, when the 3 CBCT modalities are utilized in combination, the diagnostic yield is significantly superior to that of dental radiography.

Considerations for the use of DR vs. CBCT imaging as a primary diagnostic modality, in addition to differences in diagnostic yield, include image acquisition time, necessity for general anesthesia and associated patient risks, and radiation exposure (14, 20). There is a substantial difference in time for image acquisition of a complete study, with image acquisition for CBCT taking approximately 24 s. Comparably, the efficiency in obtaining a diagnostic series of dental radiographs for a cat is dependent on having skilled personnel trained in and proficient at the require radiographic techniques. Dental radiographs must be acquired under general anesthesia, and while CBCT scans in this study were obtained under general anesthesia, CBCT has the added benefit that acquisition may be performed, with an otherwise healthy patient under heavy sedation, rather than under full general anesthesia. Arguably, it is also worth considering utilizing CBCT imaging under reversible sedation for animals with comorbidities that place them at increased risk under general anesthesia as a means of reducing anesthetic time.

This study has few limitations of note. Selection of certain anatomic structures may seem like a source of potential bias; however, these structures were selected because they are considered clinically relevant. The statistical methodology utilized in this study (Wilcoxon signed rank test) is most accurate with paired data sets larger than the one presented here, however appropriate adjustments (Bonferroni–Holm multiple comparison adjustment) were made to offset this limitation. Additionally, the concept of generalizability, which describes the extent to which research findings can be applied to settings other than that in which they were originally tested, must be taken into consideration regarding future directions in this line of research. Importantly, recognition that there are differences in diagnostic yield between image viewing methods for a single imaging modality such as CBCT emphasizes the importance of developing standardized orientation and viewing protocols (21–23). Also, the training and use of multiple evaluators could lead to more accurate results (23–25). Multiple evaluators would have allowed for inter-observer variability to be determined, reduced the possibility of interpretation bias, and strengthened the study overall.

Finally, the results of this study have immediate implications for clinical practice. This study establishes CBCT as the diagnostic criterion-reference standard for identifying anatomic structures in cats and can now serve as the foundation on which to perform a diagnostic accuracy study evaluating dentoalveolar lesions in cats. Additionally, the superior information obtained by CBCT imaging will allow for improvements in maxillofacial trauma management and surgical planning. Endodontic and orthodontic treatment planning as well as maxillofacial surgical planning are all commonly done in human dentistry utilizing CBCT technology (26–30). Future descriptive studies classifying maxillofacial fracture trauma, in addition to descriptive endodontic and orthodontic studies utilizing CBCT imaging will be able to refer to this study to be able to confidently identify these anatomic structures and utilize them as descriptive landmarks.

In conclusion, the goal of the study reported here was to semi-quantitatively assess the ability to use conventional DR and CBCT and 3 software modules to identify important anatomic structures in cats and to compare these to DR. For the conditions of this study, it can be concluded that our hypothesis was confirmed, and the results of this study may promote the utilization of CBCT technology in veterinary dentistry.

## Author Contributions

CH: study concept and design, acquisition of data, analysis and interpretation of data, and drafting of the manuscript; BA and FV: study concept and design, analysis and interpretation of data, critical revision of the manuscript for important intellectual content, administrative, technical, or material support, and study supervision; obtaining funding; DH: critical revision of the manuscript for important intellectual content; PK: statistical analysis; FV: had full access to all of the data in the study and takes responsibility for the integrity of the data and the accuracy of the data analysis.

### Conflict of Interest Statement

The authors declare that the research was conducted in the absence of any commercial or financial relationships that could be construed as a potential conflict of interest.
